# On the Action of Cyclosporine A, Rapamycin and Tacrolimus on *M. avium* Including Subspecies *paratuberculosis*

**DOI:** 10.1371/journal.pone.0002496

**Published:** 2008-06-25

**Authors:** Robert J. Greenstein, Liya Su, Ramon A. Juste, Sheldon T. Brown

**Affiliations:** 1 Department of Surgery, Veterans Affairs Medical Center (VAMC), Bronx, New York, United States of America; 2 Laboratory of Molecular Surgical Research, Veterans Affairs Medical Center (VAMC), Bronx, New York, United States of America; 3 Department of Medicine, Veterans Affairs Medical Center (VAMC), Bronx, New York, United States of America; 4 Institution Vasco de Investigation y Desarrollo Agrario Berreaga, Derio, Spain; The Research Institute for Children at Children's Hospital New Orleans, United States of America

## Abstract

**Background:**

*Mycobacterium avium* subspecies *paratuberculosis* (MAP) may be zoonotic. Recently the “immuno-modulators” methotrexate, azathioprine and 6-MP and the “anti-inflammatory” 5-ASA have been shown to inhibit MAP growth *in vitro.* We concluded that their most plausible mechanism of action is as antiMAP antibiotics. The “immunosuppressants” Cyclosporine A, Rapamycin and Tacrolimus (FK 506) treat a variety of “autoimmune” and “inflammatory” diseases. Rapamycin and Tacrolimus are macrolides. We hypothesized that their mode of action may simply be to inhibit MAP growth.

**Methodology:**

The effect on radiometric MAP ^14^CO_2_ growth kinetics of Cyclosporine A, Rapamycin and Tacrolimus on MAP cultured from humans (Dominic & UCF 4) or ruminants (ATCC 19698 & 303) and *M. avium* subspecies *avium* (ATCC 25291 & 101) are presented as “percent decrease in cumulative GI” (%-ΔcGI.)

**Principal Findings:**

The positive control clofazimine has 99%-ΔcGI at 0.5 µg/ml (Dominic). Phthalimide, a negative control has no dose dependent inhibition on any strain. Against MAP there is dose dependent inhibition by the immunosuppressants. Cyclosporine has 97%-ΔcGI by 32 µg/ml (Dominic), Rapamycin has 74%-ΔcGI by 64 µg/ml (UCF 4) and Tacrolimus 43%-ΔcGI by 64 µg/ml (UCF 4)

**Conclusions:**

We show heretofore-undescribed inhibition of MAP growth *in vitro* by “immunosuppressants;” the cyclic undecapeptide Cyclosporine A, and the macrolides Rapamycin and Tacrolimus. These data are compatible with our thesis that, unknowingly, the medical profession has been treating MAP infections since 1942 when 5-ASA and subsequently azathioprine, 6-MP and methotrexate were introduced in the therapy of some “autoimmune” and “inflammatory” diseases.

## Introduction

The “immunosuppressants” Cyclosporine A [1], Rapamycin [2] and Tacrolimus (FK 506) [3] have conventionally been used to prevent or treat the rejection of transplanted organs.[4]–[8] They have well described mechanisms of actions [9], [10] including calcineurin phosphatase inhibition by Cyclosporine and Tacrolimus and cell cycle inhibition by Rapamycin.[11] These agents are also used in the therapy of a variety of “autoimmune” and “inflammatory” diseases including inflammatory bowl disease (IBD) [12]–[19], skin diseases [20], asthma [21] and rheumatoid arthritis.[22], [23] Generally, the effect of these immunosuppressants has been studied on intact animals or eukaryotic cells, although the effect on viruses has been addressed.[24]

*M. avium* subspecies *paratuberculosis* (MAP) causes a chronic wasting enteritis in ruminants called Johne's disease [25] that is highly evocative of Crohn's disease (CD.) [26] MAP has been cultured from USA chlorinated potable municipal water [27], pasteurized milk in the USA [28], and Europe [29] [30], breast milk of mothers with CD [31] and from the blood of patients with IBD. [32] Although controversial, there are increasingly compelling data [27], [32]–[35] (& see [36] for review) that *Mycobacterium avium* subspecies *paratuberculosis* (MAP) may be zoonotic. [33]

Until recently, it was unrecognized that the “anti-inflammatory” 5 amino salicylic acid (5-ASA) [37] and the “immune modulators” methotrexate [34], azathioprine [38] and its metabolite 6-mercapto-purine (6-MP) [34], [38] are antiMAP antibiotics. Antecedent studies evaluating the potential zoonotic character of MAP had permitted these “anti-inflammatory” and “immune-modulating” agents to be used in the control groups, as their antiMAP activity was not appreciated. We therefore concluded that all those prior studies now need to be reevaluated, as their control groups were not placebo. [34], [37] Nevertheless, prevailing medical dogma [39] considers that MAP is not zoonotic.

It is of considerable interest that all three “immunosuppressants” were isolated from fungi, the source of multiple antibacterial antibiotics. Cyclosporine A, a cyclic undecapeptide, has immunosuppressant, anti-rheumatic [40], [41], dermatological [42] and anti-asthmatic [21] activity. Tacrolimus [3], [20] and Rapamycin [2], [20] are from the macrolide antibiotic family of medications, amongst the most potent anti *M. avium* antibiotic families. [43]

We hypothesized that in addition to their protean effects on eukaryotes [9]–[11], [44]–[46], and fungi [47], Cyclosporine A, Rapamycin and Tacrolimus, may also effect prokaryotes. Specifically we hypothesized that they would have antiMAP antibiotic activity. Accordingly, in bacterial culture we evaluate the effect of Cyclosporine A, Rapamycin and Tacrolimus on *M. avium,* including its subspecies MAP.

## Methods

This study was approved by the Research & Development Committee at the VAMC Bronx NY (0720-06-038) and was conducted under the Institutional Radioactive Materials Permit (#31-00636-07).

### Bacterial Culture

In this study we studied six strains of mycobacteria, four of which were MAP. Two MAP strains had been isolated from humans with Crohn's disease. Dominic (ATCC 43545, originally isolated by R. Chiodini from the intestine of a patient with Crohn's disease [48]) and UCF 4 (gift of Saleh Naser UCF Orlando FL., originally cultured from the blood of a patient with Crohn's disease.)[32] The other two MAP strains were from ruminants with Johne's disease ATCC 19698 (ATCC Rockville MD) and 303 (gift of Michael Collins Madison WI.) The *M. avium* subspecies *avium* strains (hereinafter called *M. avium*) were ATCC 25291 (veterinary source) and *M. avium* 101. [49]

Because it renders clinically resistant strains of MAP inappropriately susceptible to antimicrobials in cell culture, [50] we did not use the detergent Tween 80 (recommended to prevent mycobacterial clumping) in culture. Prior to inoculation, cultures were processed as described. [34], [37], [51]

In this study, for experimental comparability we used chemicals that could be solubilized with DMSO (Sigma St Louis MO.) The positive control antibiotic was clofazimine (an antibiotic used to treat leprosy [52] and now in clinical trials against Crohn's disease [39], [53].) The two negative controls are the gluterimide antibiotics, cycloheximide and phthalimide.

The tested agents Cyclosporine A, Rapamycin and Tacrolimus (Sigma & LC Labs. Woburn MA) were solubilized in 100% DMSO. Aliquots were prediluted, stored at −80°C in 50% DMSO (Sigma) & 50% water, thawed, used once and discarded. Volumes of DMSO were adjusted so that final concentration in every Bactec vial used was always 3.2% DMSO. Agents were tested in serial dilutions from a minimum of 0.5 µg/ml to a maximum of 64 µg/ml (see individual Figures & Tables). Inhibition of mycobacterial growth is expressed as % -ΔcGI, and enhancement as % +ΔcGI compared to 3.2% DMSO controls. [37]

**Figure 1:**
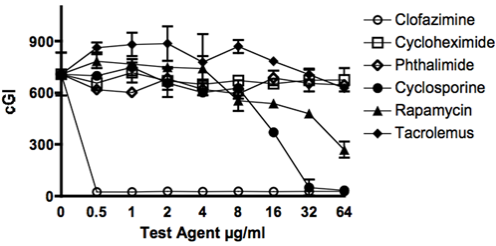
Shown are the inhibition data for a study employing MAP Dominic. The agents evaluated are Cyclosporine, Rapamycin and Tacrolimus. Of these three agents, the most pronounced inhibition is observed with Cyclosporine (see also Table 4.) Error bars are ±SD. cGI = cumulative Growth Index (Bactec®) The GI was 240 at the time of passage.

**Figure 2 pone-0002496-g002:**
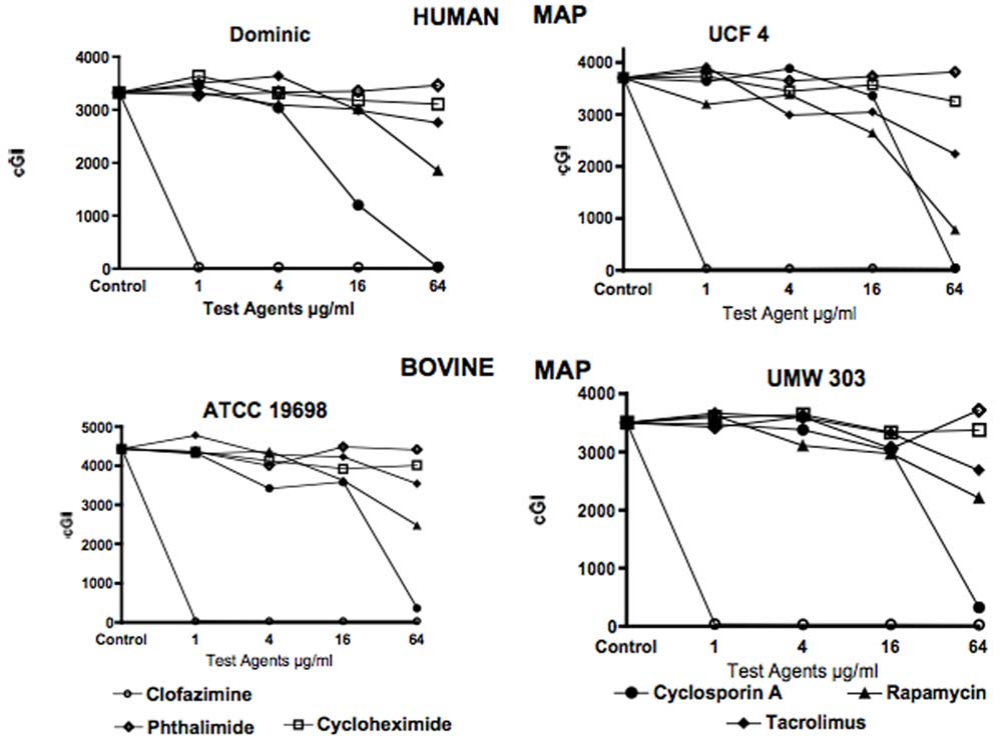
Shown is a composite of four MAP strains. The upper two are MAP isolated from humans, the lower two, MAP isolated from ruminants. “UMW 303” is University of Madison Wisconsin. UCF -4 is University of Central Florida. Note how cyclosporine is consistently the most effective of the three “immunosuppressants” tested (see also Table 5) followed by Rapamycin. The least effective of the three macrolides is Tacrolimus. cGI = cumulative Growth Index (Bactec®) For Dominic the GI was 331 at the time of passage.

**Figure 3:**
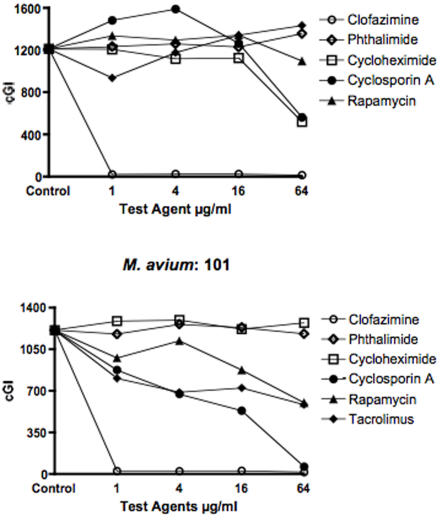
Shown is a composite of two *M. avium* subspecies *avium* strains ATCC 25291 & 101. Tacrolimus has most inhibition on *M. avium* 101 but enhances growth on *M. avium* ATCC 25291 cGI = cumulative Growth Index (Bactec®).

Data are presented in two ways: For individual mycobacterial strains as graphs (MAP in Figures 1& 2, and *M. avium* in Figure 3.) For individual chemical agents data are presented in tabular form. The positive experimental control is clofazimine (Table 1.) The “negative” controls are cycloheximide (Table 2) and phthalimide (Table 3.) Data for the “immunosuppressives” are Cyclosporine A (Table 4), Rapamycin (Table 5) and Tacrolimus (Table 6.)

**Table 1:** %-ΔcGI Clofazimine.

µg/ml	Mycobacterial strain						
	M. avium subspecies paratuberculosis (MAP)					M. avium	
	Human MAP			Bovine MAP		Bovine	
	Dominic		UCF 4	303	19698	25291	101
	Fig. 1	Fig. 2	Fig. 2	Fig. 2	Fig. 2	Fig. 3	Fig. 3
1	−99%	−99%	−99%	−99%	−99%	−98%	−98%
4	−99%	−99%	−99%	−99%	−99%	−98%	−98%
16	−99%	−99%	−99%	−99%	−99%	−98%	−98%
64	−99%	−99%	−99%	−99%	−99%	−99%	−99%

%-ΔcGI = percent decrease in cumulative GI compared to control inoculation.

**Table 2:** %-ΔcGI Cycloheximide.

µg/ml	Mycobacterial strain						
	M. avium subspecies paratuberculosis (MAP)					M. avium	
	Human MAP			Bovine MAP		Bovine	
	Dominic		UCF 4	303	19698	25291	101
	Fig. 1	Fig. 2	Fig. 2	Fig. 2	Fig. 2	Fig. 3	Fig. 3
1	15%	9%	1%	3%	−2%	0%	6%
4	−4%	0%	−7%	4%	−7%	−8%	7%
16	−8%	−4%	−4%	−5%	−12%	−7%	1%
64	−1%	−7%	−12%	−4%	−9%	−57%	5%

%-ΔcGI = percent decrease in cumulative GI compared to control inoculation.

**Table 3:** %-ΔcGI Phthalimide.

µg/ml	Mycobacterial strain						
	M. avium subspecies paratuberculosis (MAP)					M. avium	
	Human MAP			Bovine MAP		Bovine	
	Dominic		UCF 4	303	19698	25291	101
	Fig. 1	Fig. 2	Fig. 2	Fig. 2	Fig. 2	Fig. 3	Fig. 3
1	−3%	−1%	4%	−2%	−2%	2%	−3%
4	7%	0%	−2%	3%	−10%	4%	4%
16	0%	1%	1%	−13%	1%	1%	2%
64	2%	4%	3%	6%	0%	12%	−2%

%-ΔcGI = percent decrease in cumulative GI compared to control inoculation.

**Table 4:** %-ΔcGI Cyclosporine A.

µg/ml	Mycobacterial strain						
	M. avium subspecies paratuberculosis (MAP)					M. avium	
	Human MAP			Bovine MAP		Bovine	
	Dominic		UCF 4	303	19698	25291	101
	Fig. 1	Fig. 2	Fig. 2	Fig. 2	Fig. 2	Fig. 3	Fig. 3
1	15%	4%	−2%	−1%	−2%	22%	−28%
4	−10%	−9%	5%	−3%	−23%	31%	−44%
16	−43%	−64%	−9%	−14%	−19%	4%	−56%
64	−98%	−99%	−99%	−91%	−92%	−54%	−95%

%-ΔcGI = percent decrease in cumulative GI compared to control inoculation.

**Table 5:** %-ΔcGI Rapamycin.

µg/ml	Mycobacterial strain						
	M. avium subspecies paratuberculosis (MAP)					M. avium	
	Human MAP			Bovine MAP		Bovine	
	Dominic		UCF 4	303	19698	25291	101
	Fig. 1	Fig. 2	Fig. 2	Fig. 2	Fig. 2	Fig. 3	Fig. 3
1	21%	0%	−14%	4%	−3%	10%	−19%
4	13%	−7%	−9%	−11%	−1%	7%	−7%
16	−10%	−9%	−29%	−15%	−18%	11%	−28%
64	−58%	−44%	−76%	−39%	−43%	−18%	−39%

%-ΔcGI = percent decrease in cumulative GI compared to control inoculation.

**Table 6:** %-ΔcGI Tacrolimus.

µg/ml	Mycobacterial strain						
	M. avium subspecies paratuberculosis (MAP)					M. avium	
	Human MAP			Bovine MAP		Bovine	
	Dominic		UCF 4	303	19698	25291	101
	Fig. 1	Fig. 2	Fig. 2	Fig. 2	Fig. 2	Fig. 3	Fig. 3
1	28%	5%	6%	5%	8%	−23%	−34%
4	9%	9%	−19%	3%	−3%	−3%	−43%
16	21%	−10%	−18%	−5%	−5%	11%	−40%
64	0%	−21%	−43%	−27%	−26%	53%	−52%

%-ΔcGI = percent decrease in cumulative GI compared to control inoculation.

In Table 7 we present the “High” trough doses of the three immunosuppressives that are used to treat organ transplant rejection in eukaryotes. These are compared with the “Low” dose that are used to treat “inflammatory” diseases and that we posit are actually treating a prokaryote (specifically we suggest a MAP) infection.

**Table 7:** Immunosuppressant Therapeutic Trough levels used in “High” dose for transplantation rejection and “Low” dose in “Inflammatory” Diseases.

Medication	Targeted Trough levels (ng/ml)			
	“High” level for Organ Transplant Rejection		“Low” level in “Inflammatory” Diseases	
	ng/ml	Citation	ng/ml	Citation
Cyclosporine A	100–400	[63]	70–130	[14]
	396	[64]	100–200	[12]
	350–400	[65]		
Tacrolimus (FK 506)	5–20	[63]	4–8	[67]
	17–18	[66]	5–10	[68]
Rapamycin	5–15	[63]	No data available (PubMed)	

## Results

The most potent positive control is clofazimine, 97% −ΔcGI at 0.5 (Dominic; Figure 1 & Table 1.) The negative controls chemical agents are the gluterimide antibiotics cycloheximide and phthalimide. Cycloheximide has no dose dependent inhibition on any MAP strain (Figures 1 & 2 & Table 2.) Cycloheximide has dose dependent inhibition on *M. avium* ATCC 25291, (57% −ΔcGI at 64 µg/ml) but no effect on *M. avium* 101 (Figure 3 & Table 2.) Phthalimide, has no dose dependent effect on any strain tested (Figures 1–3 and Table 3.)

The three “Immunosuppressants” tested were Cyclosporine A, Rapamycin and Tacrolimus. There are differing amounts of inhibition depending on the agent and strain.

The control mycobacterial strains are *M. avium* subspecies *avium* ATCC 25291 and 101. Of the three “Immunosuppressants,” Cyclosporine A has dose dependent inhibition on *M. avium* subspecies *avium* 101 (95% −ΔcGI at 64 µg/ml) (Figure 3 and Table 4.) There is no inhibition with Rapamycin or Tacrolimus on the control *M. avium* 25291 (Figure 3 and Table 5 & 6.)

Against MAP, Cyclosporine A is the most effective of the three “immunosuppressants” studied. On MAP isolated from humans, (Dominic and UCF 4), Cyclosporine has 97% −ΔcGI at 32 µg/ml against Dominic (Figure 1) and 99% −ΔcGI at 64 µg/ml on Dominic and UCF 4 (Figure 2 & Table 4.) On MAP isolated from ruminants, Cyclosporine A has slightly less dose dependent inhibition (ATCC 19698: 92% −ΔcGI at 64 µg/ml) than against MAP isolated from humans (Figure 2 & Table 4.)

Rapamycin is the second most effective “immunosuppressant” studied. At lower concentrations (1 & 16 µg/ml) Rapamycin has no inhibition and by 64 µg it has 76% −ΔcGI on UCF 4, a MAP isolated from humans (Figure 2 & Table 5). Rapamycin is less effective against MAP isolated from ruminants and has no effect on *M. avium* ATCC 25291 (Figure 3 & Table 5.)

Tacrolimus has the least inhibition of the three “immunosuppressants” studied. Against MAP, Tacrolimus is most inhibitory against UCF 4 (43% −ΔcGI at 64 µg/ml) and ATCC 19698: 26% −ΔcGI at 64 µg/ml) (Figures 1 & 2 and Table 6.) Paradoxically, Tacrolimus exhibits the most inhibition on *M. avium* 101 of all six strains studied, yet actually enhances growth on *M. avium* ATCC 25291. (Figure 3 and Table 6.)

## Discussion

Rapamycin was initially evaluated as an anti-fungal agent. [54] To our knowledge however, this is the first time that antiMAP activity has been demonstrated for the “immunosuppressant” agents Cyclosporine, Rapamycin and Tacrolimus. These observations are therefore compatible with our thesis that MAP may be responsible for multiple “autoimmune” and “inflammatory” diseases, and that the action of these three “immunosuppressant” agents may simply be to inhibit MAP growth.

We have observed that methotrexate and 6-MP are used in “high” doses to treat human malignancies and at “low” doses in “autoimmune” and “inflammatory” conditions. [34] Similarly, there are “high” and “low” doses of the three “immunosuppressants” we now study (See Table 7.) The “high” doses are used to prevent or treat transplanted organ rejection. The “low” doses are used to treat “autoimmune” and “inflammatory” diseases. These data are compatible with our hypothesis that Cyclosporine, a cyclic undecapeptide, as well as Rapamycin and Tacrolimus, from the macrolide family of antibiotics, may have “low” dose prokaryotic antibiotic action in addition to “high” dose eukaryotic immunosuppressant activity.

Our observations are subtle and the negative controls are critical. For those not conversant with quantifying mycobacterial growth and determining the inhibitory effect of various agents, it must be emphasized that these data were obtained using the exquisitely sensitive radiometric ^14^C Bactec system®. Just as with 5-ASA [37], [38], these effects may not be detectable using the more convenient, fluorescent based MIGT system®.

The chronic use of antibiotics, even for complex mycobacterial diseases, is not advocated. With leprosy the WHO recommends that MDT be limited to ≤2 years [52] and for tuberculosis ≤18 months and preferably six months. [55] The “immunosuppressant, ” “antiinflammatory” and “immunomodulatory” agents that we show are antiMAP antibiotics have been administered indefinitely.

In the event that MAP is accepted as being zoonotic, there will need to be a reevaluation of how best to manage MAP infections in humans. There will be multiple factors that will then need to be taken into consideration. These include the fact that successfully treated leprosy and tuberculosis infections do not lead to mycobacterial eradication. Often the bacteria merely enter into a quiescent or “latent” phase and clinical symptoms progress [56] despite apparently “adequate” therapy. It will also be necessary to prevent reinfection, by removing MAP from the water supply [27], and food chain.[28] Genetic defects [57]–[59] that predispose to MAP infections will need to be identified, as affected individuals may need life long antiMAP therapy. Optimal MAP antibiotic combinations will need to be established. Designing clinical trial that consider the recently described antiMAP activity of “antiinflammatories”, “immunomodulators” and “immunosuppressants” will need to be performed. Finally, the role of MAP pre and post exposure vaccination will need to be addressed. [60]–[62]
